# New Appliances and Things Medical

**Published:** 1894-10-20

**Authors:** 


### NEW APPLIANCES AND THINGS MEDICAL.

### THE AYLESBURY DAIRY COMPANY'S PREPARATIONS.

(The Aylesbury Dairy Company, Limited, 31, St. Petersburg Place, Bayswater, London, W.)

We have received a case of samples of the milk preparations which this firm are at present placing on the market, and have made an examination of some of them. It is well known that this company enjoys a high reputation for the excellency of its milk products, and has been fortunate for some years past in having in its employ chemists of known reputation who control their work.

These preparations, with the exception of the Koumiss, are contained in specially-constructed bottles, in which the milk is sterilised and then hermetically sealed. In this way the absence of micro-organisms is ensured, and the milk can be kept for any length of time without undergoing any fermentative change. Unfortunately, the heating of the milk brings about a partial separation of the fat from the liquid, and this to some extent decreases the value of these preparations. This is to be regretted, inasmuch as nurses and mothers using the humanised milk and special milk food for infants are likely to use the liquid without mixing it with the separated fat. We note that the company do not heat to the same temperature as previously, and in this way they have prevented the change in the taste and flavour of the milk which was noticed in their earlier attempts at sterilisation.

All the samples we received were perfectly sweet, and showed that the sterilization had been properly carried out.

We have made a further examination of the company's special Milk Food for Infants, which we find contains total solids amounting to 10·35 per cent., and contains therefore a less amount than that obtained in ordinary cow's milk. The percentage of casein is stated to be considerably less, while the quantity of fat and sugar remains about the same. It should find a ready sale for cases in which careful dieting is indicated.

The Peptonized Milk manufactured for invalids and sufferers from dyspepsia we find also to be perfectly sterilized and gives the Biuret reaction very markedly with copper sulphate. It was found, however, that when 50 cc's of the milk were dialysed into 100 cc's of distilled water that samples of the dialysed liquid examined after an interval of ten minutes did not give any Biuret reaction; it follows, therefore, that although the milk has been partially digested the albumen has not been converted into peptones but remains in some intermediate albumose condition. We should prefer to call this milk pre-digested rather than peptonized, since the term peptones is now better restricted to those albuminoids which quickly diffuse through membranes.

The Koumiss manufactured by this company is supplied in four varieties, full, medium, whey, and diabetic, and varies in its composition according to its age and the temperature at which it is kept. The samples we received were labelled "Full, No. 2," and were manufactured on August 16th, and we examined them on September 26th. It was in a highly effervescent condition, and was strongly acid.

We also received a bottle of their Mouffler's Sterilised Food for infants and invalids. This solid food is also sold in vacuum stoppered bottles and contains milk, wheat flour, and eggs. We find that it contains a considerable quantity of nitrogen amounting to 2·45 per cent., which calculated to albuminoids amounts to 15·31 per cent. of these substances present. The inorganic matter amounts to 2·42 per cent. according to our analysis, and these figures confirm the composition of the food as certified by Dr. Stutzer, of Bonn, and Mr H. Droop, Richmond, the Aylesbury Dairy Company's resident analyst. It can be confidently recommended as a useful addition to our list of foods for infants and invalids.

The company's Humanized Milk is also a well prepared liquid, and has the advantage over other similar preparations in being manufactured without the addition of cane sugar. It, like the other milk preparations, is now sold in patent vacuum bottles, and, therefore, is free from any chemical preservatives like boric and salicylic acid which are frequently to be met with in commercial milk products.

The Sterilized Milk was not impaired in quality, and being preserved without the addition of chemicals is specially suitable for children and delicate persons. So long as the vacuum stoppered bottle remains unopened it remains sweet for several weeks, and is to be recommended for use on board steamers and yachts.

The specially prepared Whey is stated to be free from fat and casein, and therefore not objectionable to the most delicate digestion. It contains nearly all the salts present in whole milk, and is prepared without the addition of acid.

